# Itinerant Pile-Surgeons and Their Secret

**Published:** 1876-11

**Authors:** Edmund Andrews

**Affiliations:** Professor of Surgery in Chicago Medical College


### ITINERANT PILE-SURGEONS AND THEIR SECRET.

By EDMUND ANDREWS, A.M., M.D.,
Professor of Surgery in Chicago Medical College.

A number of men are itinerating in Illinois, and the adjacent States, and treating hæmorrhoids by a new method. The secret has been sold to various physicians and other persons, at prices varying from fifty to twelve hundred dollars, and some of the purchasers have left a good practice in the expectation of making a fortune by traveling about and applying the remedy.

The itinerants usually claim to proceed without any operative measures, but a highly intelligent physician of this State, who investigated the matter somewhat, satisfied himself that a hypodermic syringe was used, but was not certain about the fluid injection.

Subsequent investigation has placed the plan more fully in my possession, and I give it here for the benefit of all concerned.

The first thing is to have a good hypodermic syringe, kept in perfect order, with sharp, delicate pipes. The fluid used is strong carbolic acid, dissolved in any bland fixed oil. The proportions are usually as follows:

| | |
|---|---|
| R.—Crystalized carbolic acid, | oz. iij. |
| Pure oil, | f. oz. i. |

Some of the itinerants use equal parts of the two ingredients, and some of them substitute glycerine instead of oil, and at least one of them has tried a preparation of ergot.

When the piles are internal and not readily brought down, a Sims' speculum is employed to uncover them. The operator generally takes only one pile at a time, always selecting the uppermost first, and injects into its interior from four to six drops of the carbolized oil, or rather the oleized carbolic acid. The injection turns the pile white, probably coagulates the blood in its vessels, and results in its shrinking away without the inflammation being severe enough at any one time, as a general thing, to prevent the patient from attending to his business. The well-known power of carbolic acid to act as a local anæsthetic, antiphlogistic and antisupparative, favors the progress. When the irritation of the first injection has measurably subsided, another pile is attacked in the same way, and, as the patient cannot see the syringe, he supposes that he has not been subjected to any "operation," which is a great satisfaction to him. The itinerants often call their plan "painless," but it proves in some persons atrociously distressing.

The result is in many other cases excellent, so that the plan may turn out to be worthy of a permanent place in the treatment of hæmorrhoids.

However, the question whether it is perfectly safe has yet to be decided. In some instances these itinerants have gotten into an alarm at the condition of their patients, and begged earnestly for advice from men who knew more of surgery than themselves, but I have not yet heard of any actual deaths.

The injection of coagulating fluids into enlarged veins in other parts of the body has been extensively tested, the article used generally being tincture of iron. Maisonneuve, of Paris, practiced this class of injections in a great number of cases with success; but as experience accumulated increased dangers were discovered, and a number of patients have almost instantly died under the operation. The mode of death is supposed to have been this: Drops of the coagulating fluid thrown into an enlarged vein may become covered with a thin pellicle of coagulum, and in that state be swept on into the heart, where, by the bursting of the pellicle, the fluid is diffused, and a large coagulum may be instantly formed, and death by embolism occur.

If anything analogous should result from the injection of the carbolic acid and oil into the hæmorrhoidal veins, death would not be likely to occur suddenly, because these veins terminate in the portal system, and therefore any encapsuled globules or floating coagula would be arrested in their passage by the capillaries of the liver. Whether the clots thus lodged in the liver would, when large, fatally obstruct the portal vein, and when small, produce hepatitis and hepatic abscess, is a question which cannot at present be answered. It is to be desired that physicians should carefully note whether any dangerous hepatic complications are developed after this method of treating piles, and if so to report at once to the journals. Hones-surgeons will not at present, perhaps, feel justified in risking it, but these rather reckless itinerants will probably test the matter extensively, and it is our duty to observe the results. If the danger of embolism proves to be practically nothing, there is probably little else to be feared, and the operation may be a valuable addition to our resources.—*Chicago Med. Jour. and Ex.*

